# Enhanced efficiency of genetic programming toward cardiomyocyte creation through topographical cues

**DOI:** 10.1016/j.biomaterials.2015.07.063

**Published:** 2015-11

**Authors:** Constant Morez, Michela Noseda, Marta Abreu Paiva, Elisa Belian, Michael D. Schneider, Molly M. Stevens

**Affiliations:** a Department of Materials, Imperial College London, London SW7 2AZ, United Kingdom; b Department of Bioengineering, Imperial College London, London SW7 2AZ, United Kingdom; c Institute for Biomedical Engineering, Imperial College London, London SW7 2AZ, United Kingdom; d National Heart and Lung Institute, Imperial College London, London W12 0NN, United Kingdom; e British Heart Foundation Centre of Research Excellence, Imperial College London, London W12 0NN, United Kingdom

**Keywords:** Stem cell, Surface topography, Micropatterning, Cardiac tissue engineering, Cardiomyocyte

## Abstract

Generation of *de novo* cardiomyocytes through viral over-expression of key transcription factors represents a highly promising strategy for cardiac muscle tissue regeneration. Although the feasibility of cell reprogramming has been proven possible both *in vitro* and *in vivo*, the efficiency of the process remains extremely low. Here, we report a chemical-free technique in which topographical cues, more specifically parallel microgrooves, enhance the directed differentiation of cardiac progenitors into cardiomyocyte-like cells. Using a lentivirus-mediated direct reprogramming strategy for expression of Myocardin, Tbx5, and Mef2c, we showed that the microgrooved substrate provokes an increase in histone H3 acetylation (AcH3), known to be a permissive environment for reprogramming by “stemness” factors, as well as stimulation of myocardin sumoylation, a post-translational modification essential to the transcriptional function of this key co-activator. These biochemical effects mimicked those of a pharmacological histone deacetylase inhibitor, valproic acid (VPA), and like VPA markedly augmented the expression of cardiomyocyte-specific proteins by the genetically engineered cells. No instructive effect was seen in cells unresponsive to VPA. In addition, the anisotropy resulting from parallel microgrooves induced cellular alignment, mimicking the native ventricular myocardium and augmenting sarcomere organization.

## 1 Introduction

The adult mammalian heart is one of the least regenerative organs in the body with a very limited innate regenerative response [1], [2]. As a consequence, loss of cardiac muscle caused by ischemic injury (myocardial infarction) largely outmatches the heart's self-renewal capacity to create new cardiomyocytes [3]. The resulting cardiomyocyte deficit, on the order of one billion cells [3], dramatically impairs cardiac pump function and eventually leads to heart failure. With a poor prognosis at 5 years, heart failure is today one of the leading causes of morbidity and mortality in the world. For these precise reasons, generating *de novo* cardiomyocytes to replace the lost ones is of high importance.

Various strategies using cell therapy have been considered to regenerate the myocardium [2], [4] including using either: (a) endogenous cells such as bone marrow or hematopoietic stem cells, or (b) exogenous stem or progenitor cells such as embryonic stem cells [5]. Among the wide range of cell types tested, adult resident cardiac progenitors hold a particular attraction because they are present in the heart already, and hold the further advantage of having an intrinsic predisposition to differentiate into the cardiac muscle lineage, ascribed to their expression of diverse cardiac transcription factors [6], [7], [8], [9], [10].

The ground-breaking work by Yamanaka and colleagues using four genetic factors (Oct4, Klf4, Sox2, c-Myc) to reprogram somatic cells into induced pluripotent stem cells (iPSCs) resembling embryonic stem cells prompted the use of analogous cocktails to reprogram adult cells forward, instead, into lineage-specific differentiated cells [11]. Among the various forward reprogramming strategies, direct trans-differentiation to the cardiac muscle cell lineage by over-expression of key transcription factors has shown great promise for cardiac repair [12]. Both *in vitro* and *in vivo*, various cocktails have been reported to show the conversion of somatic cells directly towards a cardiomyocyte fate [13], [14], [15], [16], [17], [18], [19], [20], [21], [22], [23], [24], [25], [26], [27]. Although this finding was challenged in its early days [28], many recent papers have validated the rationale underlying this methodology in both human and mouse [12]. Notably, however, no single cocktail or reprogramming strategy has yet become a gold standard for conversion of somatic cells to cardiomyocyte-like cells. The efficiency remains extremely low, and the maturation of the induced cardiac muscle cells incomplete and elusive in culture, although higher degrees of organisation may occur after adaptation to the cardiac milieu [20].

Resistance to reprogramming is known to be attributable in large part to a cell's epigenetic characteristics [29], [30]. By stabilising one cell type, epigenetic factors control a cell's fate. Conversely, chromatin remodelling enzyme inhibitors such as valproic acid (VPA) have been widely reported to enhance cellular reprogramming dramatically, significantly improving the conversion efficiency and sometimes even supplanting some reprogramming factors [31]. A similar approach has been suggested for conversion to the cardiac muscle fate [18], [32], [33].

One of the main challenges in the field of tissue engineering resides in finding the optimal cellular micro-environment by which to control cell fate and enhance maturation [34], [35], [36], [37]. For cardiac muscle, inducing artificial alignment through *ad hoc* topographical factors or manipulating the substrate stiffness [38], [39] has been shown to greatly improve sarcomere organisation and calcium cycling in various stem cell-derived cardiomyocytes [40], [41], [42]. Interestingly, the use of various biophysical cues has also been recently reported as an efficient strategy to influence chromatin remodelling and improve cellular reprogramming, working through a substrate-induced decrease in histone acetylation and mimicking the epigenetic effect of histone deacetylase inhibitors [43], [44], [45].

As a result, we address in this work the question of whether topographical cues (parallel microgrooves) in combination with a forward reprogramming strategy (a cocktail of three cardiogenic transcription factors) could positively affect *de novo* generation and maturation of cardiomyocytes from adult heart-derived progenitor cells. Specifically, based on the prior work cited [18], [43], we hypothesized that microgrooves would likewise enhance forward programming, requiring two preconditions: that histone acetylation was in fact enhanced by grooves in the precursor cells tested, and that the cells furthermore were responsive to a pharmacological HDAC inhibitor. The predicted phenotype was seen in cells fulfilling these criteria and not in cells lacking them.

## 2 Materials and methods

### 2.1 Cell culture and reprogramming

Clones were generated as previously described [46], [47] and maintained in clonal growth medium (CGM) comprising 65% (v/v) Dulbecco's Modified Eagle's Medium/Ham F-12 (Life Technologies), 35% (v/v) Iscove's Modified Dulbecco's Medium (IMDM; Life Technologies), 3.5% (v/v) bovine growth serum (Thermo Scientific), 100 U/mL Antibiotics-Antimycotics (Life Technologies), 2 mM l-glutamine (Life Technologies), 0.1 mM β-mercaptoethanol (Sigma), 1.3% (v/v) B27 media supplement (Life Technologies), 6.5 ng/mL EGF (Petrotech), 13 ng/mL FGF (Petrotech), 0.0005 U/mL thrombin (Roche), 0.345 ng/mL cardiotrophin-1 (Cell Science). For reprogramming experiments we used a previously described protocol [46]. Briefly cells were first transduced with a lentiviral rtTA-IRES-Puro vector and subsequently selected with puromycin (Life Technologies) for 14 days. Cells were then transduced with the transcription factor-encoding lentiviral vectors: TRE-Myocd-ING, TRE-Tbx5-INR (kindly obtained from Lei Zhou, Texas Heart Institute, Houston, Texas, USA) [48] and TRE-Mef2c-ING (cDNA from BioScience LifeSciences cloned onto the Myocardin lentiviral backbone [46]). One day following transfection, CGM was changed for IMDM supplemented with 10% (v/v) bovine growth serum, 100 U/mL Antibiotics-Antimycotics, 2 mM l-glutamine, 0.1 mM β-mercaptoethanol and 1 μg/mL doxycycline (Sigma) and medium changed every 48 h for the duration of the reprogramming experiment. When cells were treated with valproic acid (Sigma) a 0.5 mM concentration was systematically used.

### 2.2 Membrane fabrication

Silicon wafers patterned with microgrooves (10 μm wide, 3 μm deep) using standard soft-lithography techniques. Briefly, photoresist (SU8-2002) was spin-coated (≈250 μm) onto a silicon wafer and a patterned photomask was used to expose the photoresist to UV light. The un-polymerized photoresist was subsequently washed away. Polydimethylsiloxane (PDMS) was then prepared according to manufacturer's protocol (Sylgard 184, Dow Corning), degassed under vacuum, spin-coated onto the patterned silicon wafers and cured at 110 °C for 40 min. The resulting micro-patterned membranes were removed from the template, thoroughly washed and plasma treated prior to UV sterilization and collagen I coating (rat tail, Life Technologies).

### 2.3 Scanning electron microscopy

Cells cultured on PDMS membranes were fixed with 3.7% (v/v) formaldehyde in PBS for 15 min, and then dehydrated by washing for 5 min in progressively higher concentrations of ethanol in water (30%, 50%, 70%, 90%, 100%x2) followed by two washes in hexamethyldisilazane for 5 min. A 10 nm thin film of Cr was deposited on the sample by sputter coating, to prevent charging. The sample was analysed at 10 KeV in a LEO 1525 FEGSEM with a secondary electron detector.

### 2.4 Edu incorporation assay

Cells were pulsed with 10 μM EdU (Invitrogen) for 2 h a day after being seeded on the various substrates. Cells were subsequently fixed with 4% paraformaldehyde and permeabilized in 0.2% (v/v) Triton X-100 before incubation with Click-iT reaction cocktail (Invitrogen). 4′,6-diamidino-2-phenylindole (DAPI) was used for nuclear staining, analysis was then performed with conventional epifluorescence microscopy.

### 2.5 Immunofluorescence, imaging and quantification

Cells were fixed in 3.7% (v/v) paraformaldehyde for 10 min, then permeabilized and blocked in PBS with 4% (v/v) bovine growth serum (Thermo Scientific) and 0.2% (v/v) Triton X-100 for 1 h, and subsequently incubated with primary antibodies for AcH3 (Millipore), Actc1 (Sigma), sarcomeric MyHC (R&D), Nppa (Millipore), Ryr2 (Sigma), Pln (Abcam) in blocking buffer overnight on a rocker at 4 °C. Secondary antibody incubation (Alexa 647, Life Technologies) was performed in blocking buffer for 1 h at room temperature. The fixed PDMS membranes were then mounted on slides in Vectashield^®^ mounting medium and analysed with confocal microcospy (Zeiss LSM-780 inverted) or conventional epifluoresence microscopy with a Zeiss Axio-Observer Z1 widefield fluorescent microscope.

High-throughput image analysis was performed with a custom made Matlab^®^ script. Briefly, cell nuclei were detected using contrast enhancement methods, then segmented with an edge detection algorithm (Canny; Matlab^®^), following which elliptical fitting was performed to compute the aspect ratio (short axis/long axis) and the angle between the long axis and the groove direction.

### 2.6 Protein extraction, immunoprecipitation and Western blotting

Cells on each membrane were lysed on ice with RIPA lysis buffer supplemented with PMSF in DMSO, a protease inhibitor cocktail in DMSO and sodium orthovanadate in water (Santa Cruz). Protein lysates were centrifuged to pellet cellular debris, and the supernatant was removed and quantified by the DC Protein Assay (Bio-Rad). Protein samples were run in SDS/PAGE and transferred to nitrocellulose membranes. Membranes were blocked with 3% non-fat milk and incubated with the primary antibodies (AcH3, Millipore; GAPDH, Santa Cruz) diluted in TBST (Tris-Buffered Saline Tween-20) buffer containing 25 mM TrisHCl (pH 7.4), 60 mM NaCl, and 0.05% (v/v) Tween-20 overnight. Infrared secondary antibody (LI-COR) incubation was then performed at room temperature for 1 h before analysis with an Odyssey Imager system (LI-COR). For immunoprecipitation studies, 20 mN NEM (Sigma) was supplemented to the RIPA lysis buffer with the previously mentioned cocktail of inhibitors. Cellular lysates were pre-cleared with protein A agarose (Santa Cruz), before a 10% input was removed and the remaining lysate incubated with SUMO-1 antibody-agarose conjugated beads (Santa Cruz). The pellet was collected and extensively washed in lysis buffer before being analysed by Western blotting with anti-myocardin antibody (Abcam).

### 2.7 RNA isolation and qRT-PCR

Cells were lysed using TRIzol^®^, and RNA subsequently extracted and purified using the PureLink RNA minikit (Life Technologies). RNA concentration and purity was determined with a Thermo Scientific Nanodrop spectrophotometer.

cDNA was then reverse transcribed from RNA using the High-capacity cDNA Reverse Transcription Kit (Applied Biosystems).

qRT-PCR were performed using custom 384-well microfluidic cards (Taqman low density array cards, Life Technologies) and an ABI PRISM 7900HT Sequence Detection System (Applied Biosystems). Data analysis was performed using DataAssist™ Software v3.01 (Applied Biosystems).

### 2.8 Statistical analysis

All statistical tests were performed with the Excel Data Analysis Toolpak (2010). All results are expressed as the mean ± standard deviation (SD). To test significance, one-way ANOVA was performed. To compare the differences between microgrooved membrane samples and flat membrane samples, we used the unpaired two-tails Student's t-test. Results for which p < 0.05 were considered statistically significant (n ≥ 3 in all experiments).

## 3 Results

### 3.1 Microgrooved substrates induce cell alignment and nuclear elongation but no spontaneous differentiation of cloned cardiac side population (SP) cells

Sca1 positive (Sca1+) cardiac progenitors have been shown to have cardiac differentiation potential *in vitro* and *in vivo*, and to be a target for the enhancement of regeneration by prostaglandin E2 [6], [7], [8], [49]. Further studies have demonstrated that a subpopulation identified by the extrusion of Hoechst dye 33342, the so-called side population (SP), are enriched for clonal growth and account for cardiac differentiation capacity [50], [51]. Thus, we used two clonally derived hematopoietic lineage-negative (Lin-), Sca1+, SP resident adult progenitor cell lines [51]. Each represents the progeny derived from an independent single cell deposited by fluorescence-activated cell sorting (FACS). We chose to use clonal derivatives to remove the heterogeneous element intrinsic to the population of Sca1+ progenitors.

Two specific SP clones were selected, clones 3 and 16, hereafter referred as SP3 and SP16 respectively. SP3 and SP16 clones resemble an incomplete form of cardiac mesoderm as shown by expression of several key cardiac transcription factors (such as Gata4/6, Hand2, T-box factors, and Mef2a) but the absence of cardiomyocyte differentiation markers (such as sarcomeric gene expression) [51]. Though sharing this general signature, the cloned SP cells possess microheterogeneities of cardiac transcription factor expression [51]. Notwithstanding these differences and the transcription factors absent from the cells, both clones exhibit multi-lineage differentiation into cardiac muscle, vascular smooth muscle, and endothelial cells after delivery to healthy or infarcted myocardium [51]. By contrast, no spontaneous differentiation *in vitro* was observed (data not shown), suggesting that *in vivo* cues, potentially including physical ones, are essential to relieve their differentiation arrest following cell grafting.

**Fig. 1:**
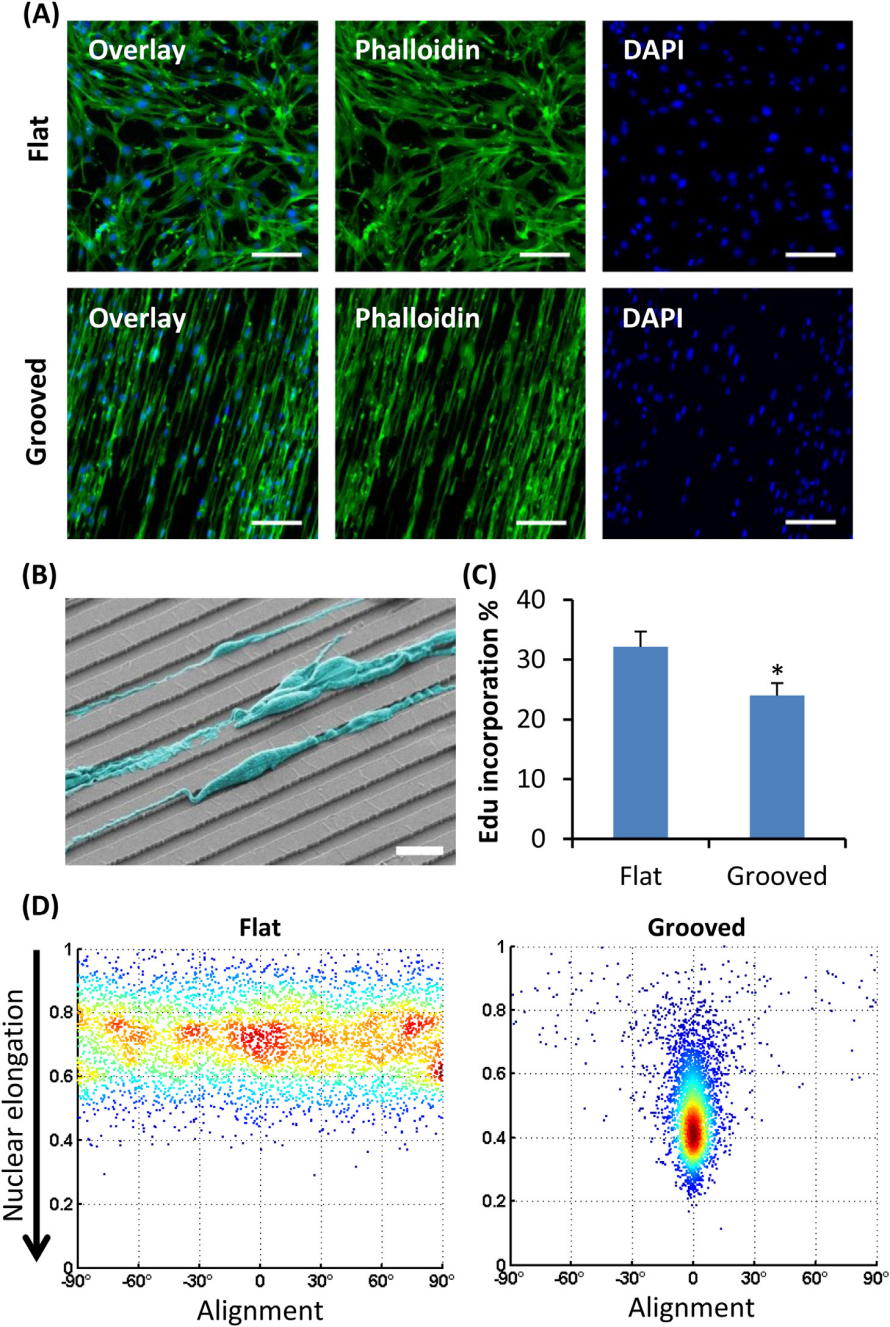
Cell and nuclear elongation in cloned cardiac stem cells on the microgrooved substrate. (A) Cytoskeletal actin (Phalloidin, green), and nuclear (DAPI, blue) staining of SP16 cells cultured for 3 days on flat and patterned substrates (scale bar: 200 μm). (B) Scanning electron microscopy of SP16 cells (cyan, pseudocolour) aligned along the grooves (scale bar: 20 μm). (C) EdU incorporation analysis showing decreased clone SP16 proliferation on grooves (n = 3, p < 0.05, error bars indicate standard deviation). (D) Scatter plot showing the nuclear elongation (aspect ratio of nuclei elliptical fitting) as a function of each cell's alignment along the grooves (angle between long axis and grooves). Cells on patterned substrates align within 10° parallel to the grooves and are significantly more elongated, 0.49 ± 0.14 on the microgrooved surface versus 0.71 ± 0.12 (p < 10^−5^). (For interpretation of the references to colour in this figure legend, the reader is referred to the web version of this article.)

The SP cells were maintained on standard tissue culture plates with collagen I coating prior to being seeded on collagen I coated polydimethylsiloxane (PDMS) membranes (Young's modulus of the order of 1 MPa) that were either flat or microgrooved (10 μm wide, 3 μm deep) and fabricated using standard soft-lithography techniques. As expected from results in other cell types [52], [53], cloned cardiac SP cells adopted a very elongated morphology on the patterned substrates as shown by cytoskeletal staining (Fig. 1A and Suppl. 1A) and scanning electron microscopy (Fig. 1B), sitting either on top of the microgrooves or falling into the ridges between the grooves. Cell proliferation, quantified by incorporation of 5-ethynyl-2’-deoxyuridine (EdU), was decreased by one-fourth on the microgrooved substrate (from 32% to 24%; Fig. 1C and Suppl1C), corroborating previous findings obtained in fibroblasts [43] or vascular stem cells [54]. Accordingly to a study positively correlating nuclear volume and DNA synthesis [55], nuclear volume on grooved substrates was significantly reduced (Fig. Suppl. 2). Notably, patterned substrates induced a dramatic nuclear elongation of both SP3 and SP16 cells (Fig. 1D and Suppl. 1B), with the average length of a cell nucleus on the grooves being twice as long as it was wide.

**Fig. 2:**
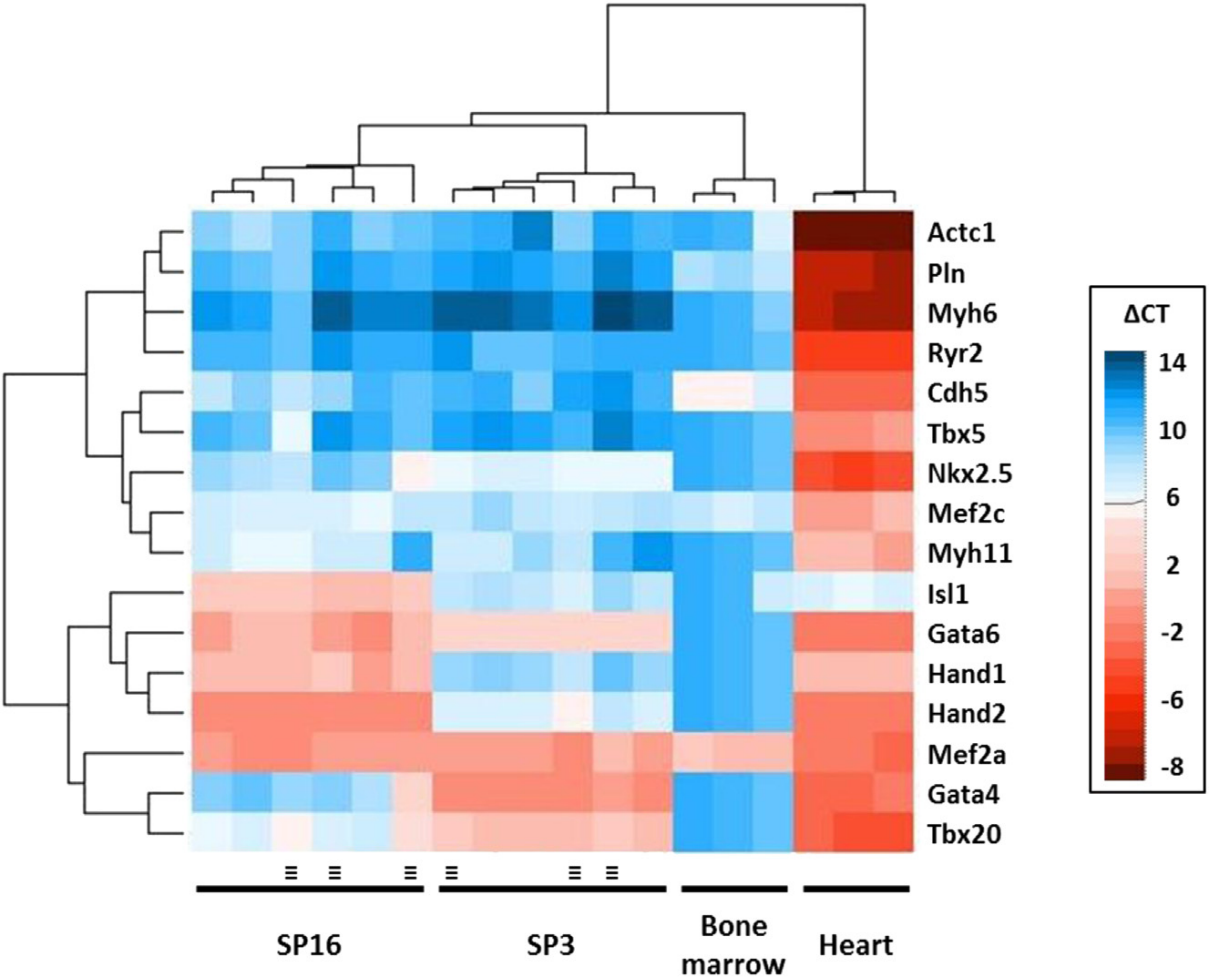
qRT-PCR heat map for genes including cardiogenic transcription factors and cardiac, smooth muscle, and endothelial differentiation markers. Clustering was performed using an unsupervised complete-linkage method for Euclidean distance. SP3, SP16, mouse heart and bone marrow controls were well resolved. No significant changes in baseline gene expression were evoked by growth on flat versus patterned (≡) membranes.

To assess the differentiation potential that results from culturing cells on the microgrooved topography, we compared the cells' phenotype after 5 days on the respective substrates. We used quantitative real-time polymerase chain reaction (qRT-PCR) to examine the expression of the indicated genes (Fig. 2), including cardiogenic transcription factors (*Gata4*, *Gata6*, *Hand1*, *Hand2*, *Mef2a*, *Mef2c*, *Nkx2.5*, *Tbx5*, *Tbx20*, *Isl1*) and representatives of the differentiated cardiac (*Actc1*, *Myh6*, *Pln*, *Ryr2*), endothelial (*Cdh5*), and smooth muscle (*Myh11*) lineages. Cardiac transcription factors and structural genes were readily detected in the adult heart (excepting *Isl1*, in keeping with its known developmental regulation) but largely absent in bone marrow as expected. The progenitor cells lack expression of cardiac or vascular structural genes but express many cardiac transcription factors suggesting they are transcriptionally predisposed to cardiac lineage differentiation but are still in an undifferentiated state. The two clones express different sets and levels of cardiac transcription factors, similar to the microheterogeneities within freshly isolated cardiac progenitor cells [51]. No genetic marker was differentially expressed more than 2-fold between flat and patterned substrates (Fig. 2). In addition, cells failed to segregate based on their culture substrate following 2-dimensional unsupervised clustering of the qPCR results (Fig. 2). Thus, no large spontaneous phenotypic changes were detected from short-term culture on a microgrooved substrate: the cell phenotype was maintained similar to that previously seen in standard cell culture conditions for these clones.

### 3.2 Histone acetylation on grooves is cell dependent and associated with nuclear elongation

**Fig. 3:**
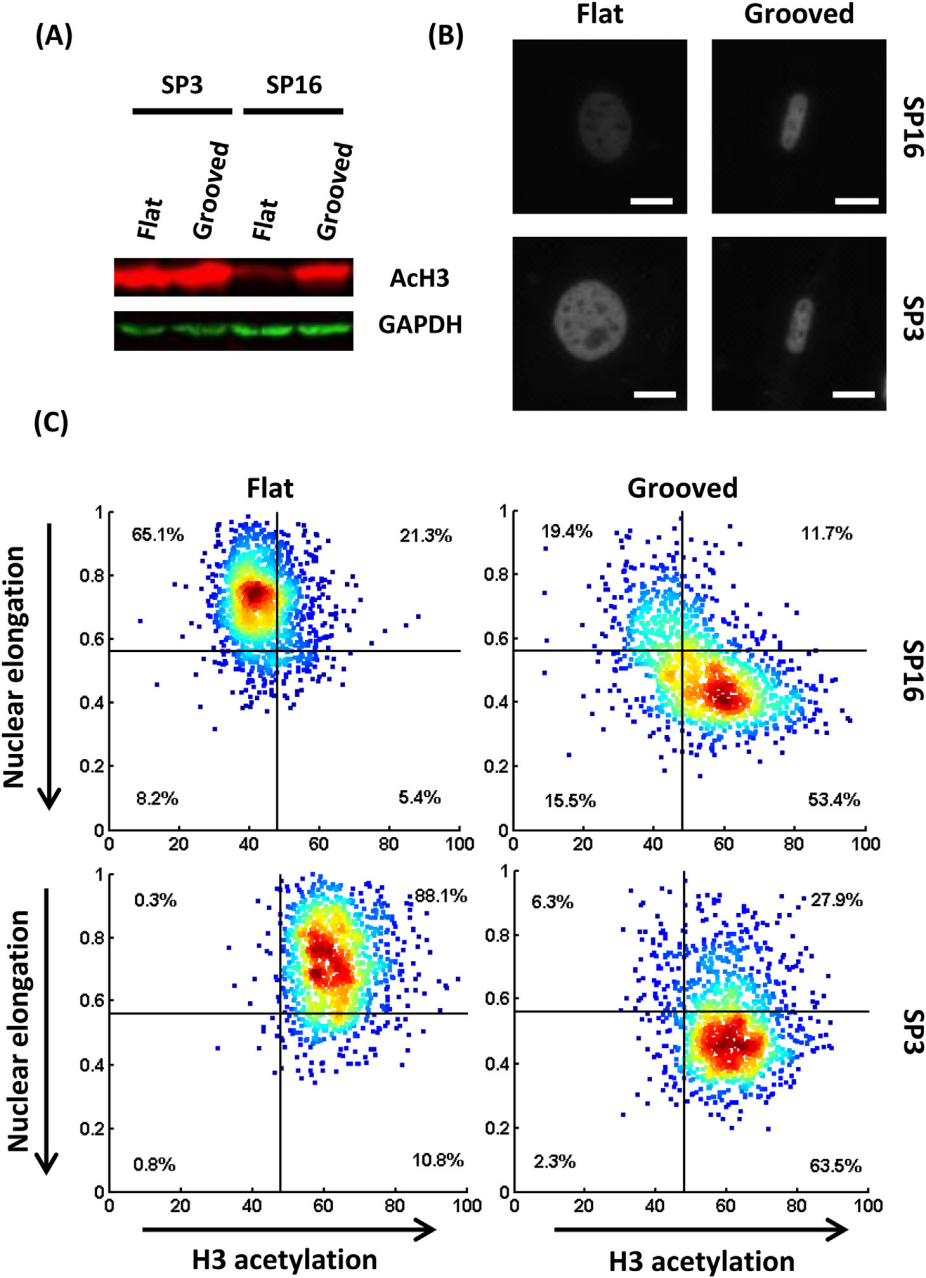
Histone 3 acetylation (AcH3) on the microgrooved substrate. (A) Western blot showing the AcH3 levels on the microgrooved versus flat substrate. (B) Representative immunostaining for AcH3 (scale bar: 20 μm). (C) Scatter plot showing AcH3 levels (immunostaining) as a function of the nuclear elongation (aspect ratio of nuclei elliptical fitting) for both clones on flat surface versus grooves. As cells' nuclei become more elongated, AcH3 levels increase on the patterned substrate in clone 16, while no further acetylation is evoked in clone 3.

Topographical cues such as parallel microgrooves have been previously reported to increase histone 3 acetylation (AcH3) in mouse fibroblasts and mesenchymal stem cells [43], [53], [56]. To test whether cloned cardiac progenitor cells were subject to the same effects, we performed Western blotting analysis and immunofluorescence microscopy. Interestingly, one of our clones, SP16, underwent an increase in AcH3 while cultured on the micropatterned substrate (Fig. 3A–B and Suppl. 3C).

By contrast, SP3 AcH3 levels on the un-patterned substrate were significantly higher than those of SP16, similar instead to SP16 cells' level of AcH3 on microgrooves (Fig. 3A–B and Suppl. 3C). This suggests that topographically induced hyper-acetylation on microgrooved substrates is not ubiquitous even within a cell type, and may depend on the cells' baseline acetylation levels. To investigate further the role of topography on histone acetylation, we looked for possible correlations between individual cells' nuclear elongation and histone acetylation, analysing thousands of nuclei with a custom Matlab^®^ script. Strikingly, we found a statistically significant direct correlation between the nuclear elongation of SP16 and levels of AcH3 (Fig. 3C and Suppl. 3A-B, p = 0.018 for a pair-wise comparison of the slopes between grooved and flat surfaces). Although, a priori, nuclear elongation might accompany increased histone acetylation rather than cause it, this finding is noteworthy in light of previous reports directly implicating the nuclear envelope protein Lamin A/C in the increase of AcH3 levels on grooved substrates [53], i.e., a nuclear basis for mechanotransduction in the setting we studied.

### 3.3 Forward programming toward the cardiomyocyte lineage is 2-fold higher on microgrooves

**Fig. 4:**
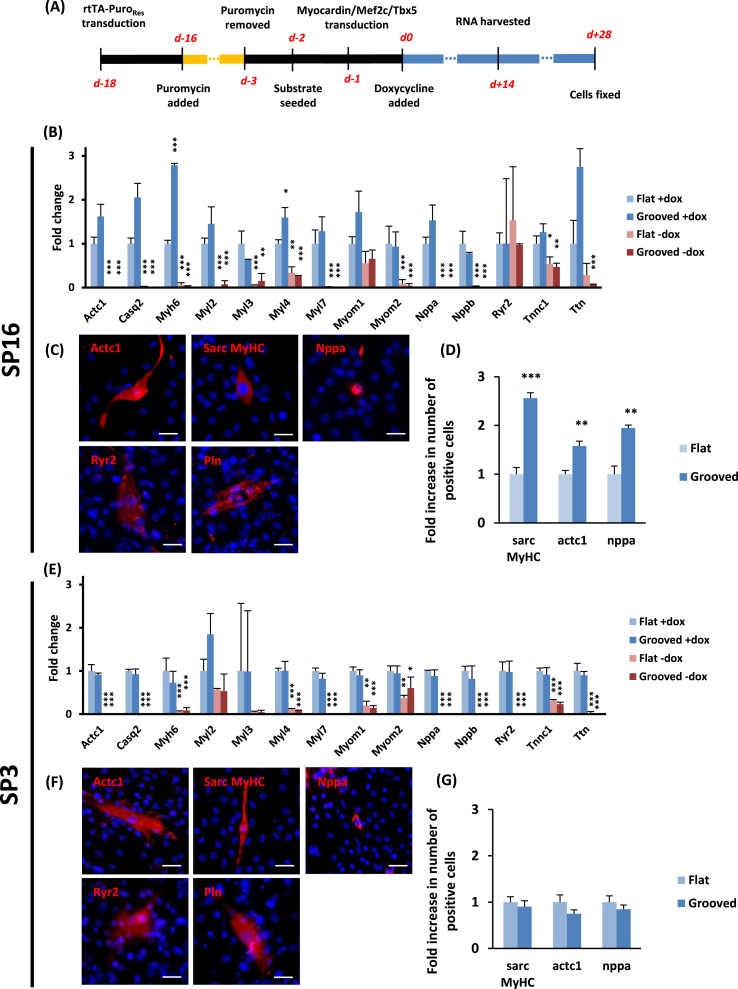
*De novo* cardiomyocyte generation (A) Schematic of the reprogramming process. (B,E) qRT-PCR results showing the induction of cardiac markers including transcription factors, sarcomeric proteins and calcium handling proteins (* indicates significance for pair-wise a comparison Student's t-test against the flat doxycycline controls, ***p < 0.001, **p < 0.01, *p < 0.05, n = 3, error bars indicate standard deviation). (C,F) Fluorescence microscopy showing reprogrammed cells on the flat surfaces positive for the indicated cardiac muscle markers (scale bar: 20 μm). (D,G) Quantification of immunostaining results (***p < 0.001, **p < 0.01, *p < 0.05; Student's t-test, n = 6, error bars indicate standard deviation).

Histone acetylation, chromatin compaction and epigenetic stability are known to be major hurdles faced in reprogramming strategies [29]. Conversely, chemical inhibitors of specific histone remodelling enzymes have been successfully used to facilitate cellular trans-differentiation by directly affecting the chromatin state [57]. For example, the histone deacetylase (HDAC) inhibitor valproic acid (VPA) has been shown to increase direct reprogramming of cardiomyocytes 2-fold (measured as sarcomeric myosin heavy chain (MyHC) positive cells) by inducing histone hyper-acetylation [18]. To assess whether our microgrooved substrates could enhance the generation of *de novo* cardiomyocytes through a biophysical regulation of histone acetylation, we infected both our SP3 and SP16 clones with a tetracycline-inducible lentiviral system, by which overexpression of a cocktail of cardiogenic transcription factors was homogeneously transduced but stringently controlled by doxycycline (Dox; Fig. 4A). Considering previously published findings [18], [24] and the expression patterns of cardiac transcription factors in our two SP clones [51], we chose to overexpress Mef2c, Myocardin and Tbx5. Fifteen days following the addition of Dox we analysed by qRT-PCR a set of 45 cardiac markers. Among those examined, a large subset was significantly up-regulated compared to the non-doxycycline control (Fig. 4B and E). These up-regulated genes comprised structural proteins (Actc1, Myh6, Myl2, Myl3, Myl4, Myl7, Myom1, Myom2, Tnnc1, Ttn) but also calcium handling proteins (Casq2, Ryr2), which indicated a cardiomyocyte-like phenotype or, at least, extensive changes toward that lineage. To further validate our findings, we performed immunofluorescent labelling for cardiac-restricted structural proteins (sarcomeric MyHC, α-cardiac actin) and calcium handling proteins (ryanodine receptor, phospholamban). Cells staining positive for those four markers were systematically found in all reprogramming experiments, using the SP3 or SP16 clones (Fig. 4C and F). Dox-dependent induction of lineage-restricted sarcomeric proteins and regulators of calcium handling is strongly supportive of progression toward a cardiomyocyte phenotype.

To test the predicted synergy between our genetic directed differentiation procedure and the microtopographical cues, we counted cells which were positive for the expression of sarcomeric MyHC, Actc1, and atrial natriuretic peptide (Nppa) concordantly. In the reprogrammed SP16 cells, we consistently observed twice as many positive cells (for all three markers) on the patterned substrate, compared to the flat one (Fig. 4D). We emphasize that the biochemical composition and stiffness of the substrates was identical, under the conditions tested, with the sole difference being the absence or presence of a microgrooved surface pattern. By contrast, consistent with the lack of change in AcH3, no difference was observed for SP3 cells (Fig. 4G), transduced under identical conditions and exhibiting comparable baseline responses to the exogenous transcription factors. Given this difference, we postulated that the observed increase in reprogramming efficiency conferred by the patterned substrate is the result of the observed increase in histone acetylation of clone SP16 on the microgrooves.

**Fig. 5:**
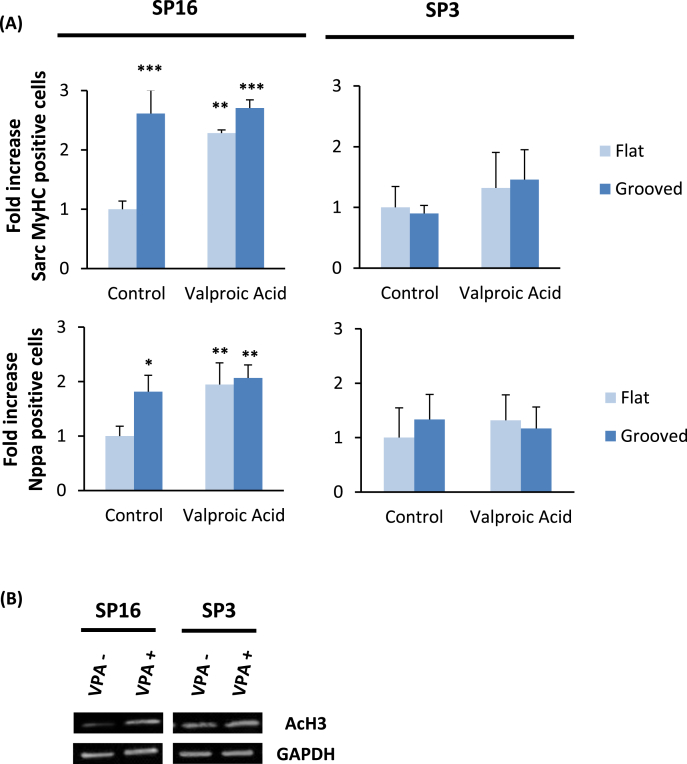
Effect of VPA-induced histone hyper-acetylation on cardiac reprogramming. (A) Sarc MyHC and Nppa expressed in transduced cells is shown as the fold increase compared to flat substrate without VPA (***p < 0.001, **p < 0.01, *p < 0.05; Student's t-test against the flat control, n = 63, error bars indicate standard deviation). (B) Western Blot showing the increase in AcH3 following incubation for 24 h with 0.5 mM VPA.

To test this hypothesis directly, we repeated the reprogramming experiments in the presence or absence of VPA, a histone deacetylase inhibitor. In transduced cells prior to the addition of doxycycline, VPA induced a global increase in AcH3 for SP16 yet no noticeable difference for SP3 (Fig. 5A). Interestingly, upon addition of doxycycline to induce the exogenous transcription factors, incubation with VPA enhanced the differentiation of SP16 cells on flat membranes, as assayed by the numbers of MyHC-positive and Nppa-positive cells, evoked no further increase in SP16 cells on microgrooved membranes, and had no effect on SP3 differentiation on either substrate (Fig. 5B). The inability of VPA to augment the reprogramming efficiency evoked by grooves suggests that the topographically-induced increased efficiency on patterned substrates could be attributable to the increased histone acetylation.

**Fig. 6:**
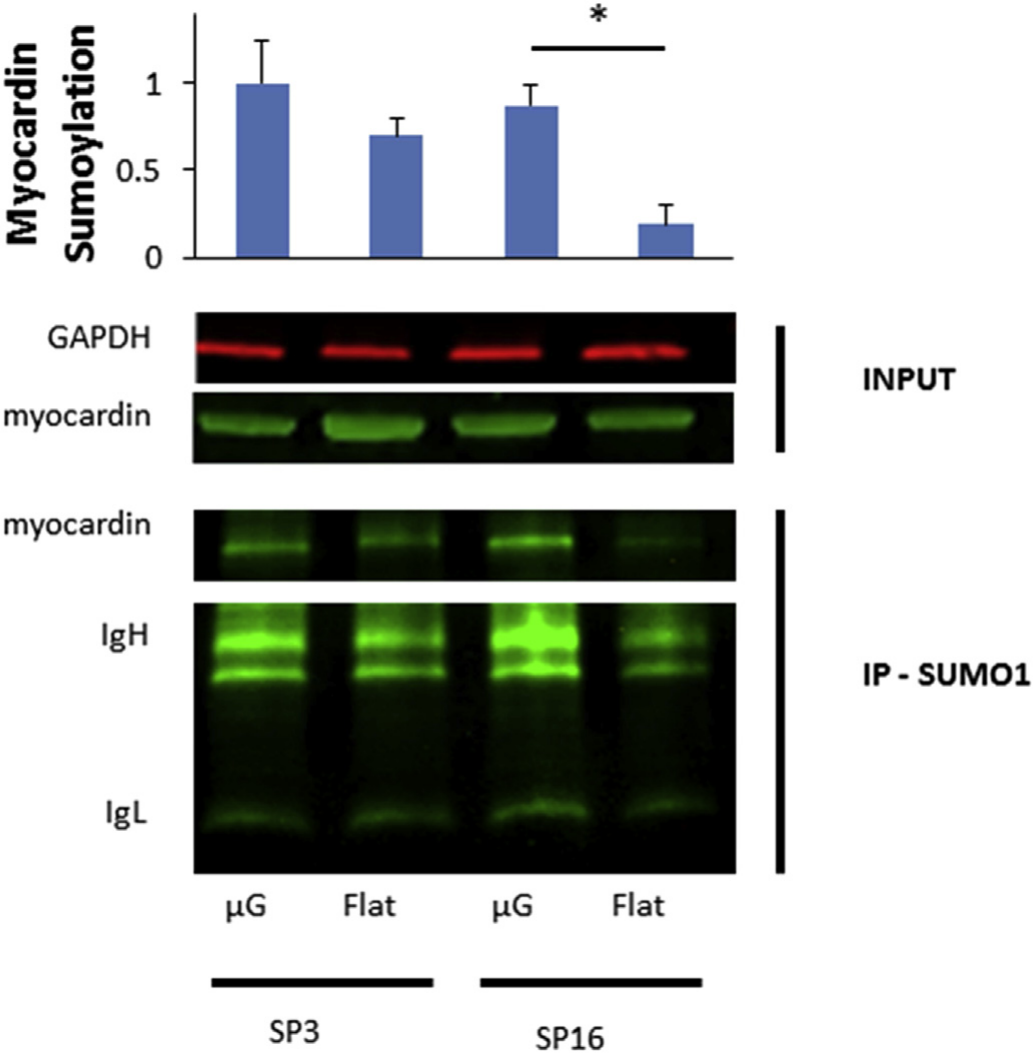
Myocardin sumoylation on flat and patterned substrates was assessed by immunoprecipitating cell lysates (INPUT) with anti SUMO-1 conjugated agarose beads (IP-SUMO). Immunoglobulin heavy (IgH) and light (IgL) chains of the IP antibody are indicated, GAPDH was used as a loading control. *p < 0.05; Student's t-test against the flat control, n = 3, error bars indicate standard deviation. Myocardin sumoylation was enhanced only in the VPA-sensitive cells (cf. Fig. 5).

To provide further evidence that increased differentiation on the microgrooved substrate is related to histone 3 acetylation and a cell's sensitivity to HDAC inhibition, we assessed the sumoylation of the cardiac co-activator myocardin, an essential component of our gene transfer study [46]. In agreement with recent reports of cardiac proteins' enhanced sumoylation after HDAC inhibition [58], we observed a significant increase of myocardin sumoylation on the microgrooved substrates, only in our HDAC inhibitor-sensitive clone SP16 (Fig. 6). Since myocardin sumoylation is proven to strongly activate cardiogenic gene activity [59], the observed differences in myocardin sumoylation provide a highly credible explanation for the increased differentiation on microgrooved substrates only in our HDAC inhibitor-sensitive cell population.

### 3.4 Sarcomere formation is enhanced on the microgrooved substrate

In addition to the mere up-regulation of cardiomyocyte-specific markers at the RNA and protein level, a further critical component of *bona fide* cardiac muscle cells is the presence of key structural attributes such as the formation of sarcomeres [12].

**Fig. 7:**
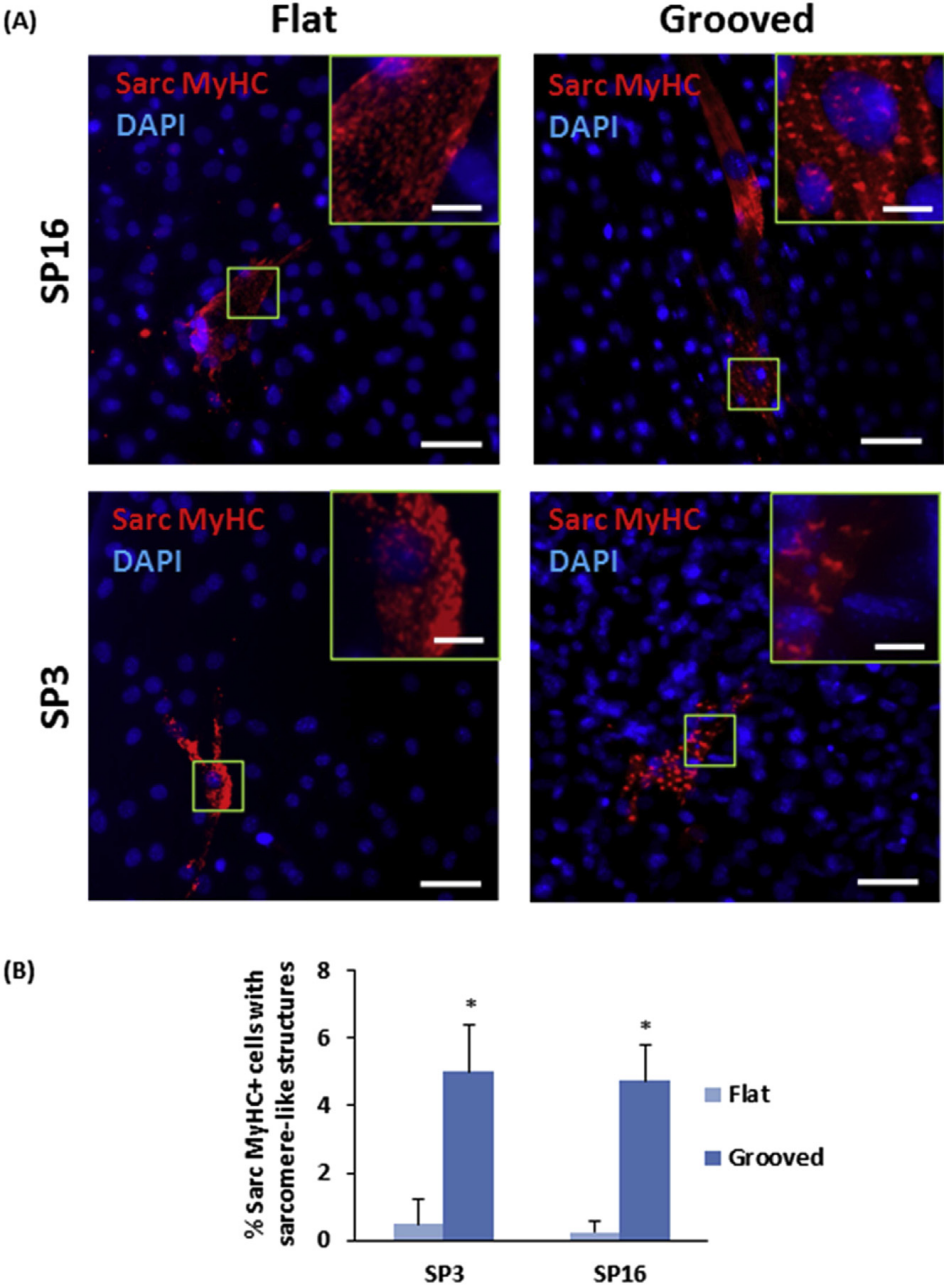
Sarcomere-like structures provoked by microgrooves. (A) Sarcomere-like structures in reprogrammed SP3 and SP16 cells, comparing grooved versus flat substrates by confocal imaging for sarc MyHC. Scale bar: 50 μm and insert, 5 μm. (B) Microgrooves induced a 10-fold increase in ordered sarcomeres (*p < 0.05; Student's t-test, n = 63, error bars indicate standard deviation).

For pluripotent cell-derived cardiomyocytes, which enter the cardiac lineage in response to developmentally relevant peptide growth factors, it has been reported that breaking the artificial *in vitro* isotropy characteristic of routine cell culture, by appropriate topographical cues, enhances the formation of sarcomeres [40]. In our reprogrammed cells, very few organized sarcomeres could be identified on the flat surfaces (Fig. 7A), suggestive of inefficient or at least incomplete reprogramming. By contrast, we found the systematic establishment of highly ordered sarcomeres on the microgrooved substrate (Fig. 7A). Quantification of such structures revealed a 10-fold increase in the number of MyHC-positive cells on the microgrooved substrate compared to the flat surface (Fig. 7B). Because sarcomere organization was enhanced equally in both lines (Fig. 7B), this aspect of the phenotype does not depend on the different baseline levels of AcH3. Conversely, no differences between the lines exist in the prevalence of sarcomere order, as a possible counter-explanation for the increased differentiation of SP16 cells on microgrooves (Fig. 5B).

## 4 Discussion

Forward reprogramming towards the cardiomyocyte lineage represents a great promise in the field of cardiac regeneration through the generation of new cardiomyocytes from differentiated cells [10]. However, low reprogramming efficiency along with imperfect differentiation remains a great challenge to overcome before forward reprogramming strategies can represent a useful source of *bona fide* cardiomyocytes. In our study, we report a novel technique, using materials engineering coupled with forward reprogramming to significantly improve the generation of *de novo* cardiomyocytes from adult cardiac progenitors both in terms of efficiency (Fig. 4D) and maturation (Fig. 7B). More specifically, we showed that the concomitant increase in AcH3 and myocardin sumoylation levels induced by the use of parallel microgrooves as topographical cues does not occur equally in all cells, yet is highly uniform within clonal derivatives of the starting cardiac progenitor cell population (Fig. 4). Our results suggest that this stable, clonally inherited difference is likely influenced by the cells' original intrinsic levels of acetylation (Fig. 4A) and can be assessed by a cell's sensitivity to HDAC inhibition (Fig. 5). In addition, we showed that the induced acetylation in susceptible cells was quantitatively related to the extent of nuclear elongation (Fig. 4C). This observation underscores the likely critical role of nuclear matrix proteins in the biophysical regulation of a cell's epigenetic landscape. Future studies will be of interest to dissect the proteins and pathways mediating mechanotransduction in this context and its epigenetic effects.

Although induced acetylation had been shown to increase reprogramming to iPSCs, allowing the chromatin to be in a state which more closely resembles that of a pluripotent stem cell [43], [60], [61], it was not clear whether the same strategy would enhance direct programming towards any differentiated lineage. In this study, we show that induced acetylation indeed can also improve forward reprogramming (Fig. 5). This result in particular opens the field to a biomaterials approach in other trans-differentiation strategies where clinical translation is critically impaired by the low efficiency associated with such strategies, and again emphasises the importance of epigenetic regulation in the control of cell fate [62]. It will be intriguing in future studies to determine whether biophysical cues act via the initial recruitment of exogenous transcription factors to their target sites, perhaps supplanting the need for pioneer factors that bind to closed chromatin, versus one or more other stages in reprogramming. Likewise, it will be of interest to dissect the signalling cascade for nanotopographical cues.

Finally, we demonstrated that in addition to enhancing the overall number of cardiomyocyte-like cells generated (Fig. 4D), the use of specific topographical cues could enhance the maturation of induced cardiomyocytes by providing guidance and cues that mimic the *in vivo* environment (Fig. 7). To our knowledge, this is the first study where a material-based approach has been used to facilitate cell fate switching through a biophysical regulation a cell's epigenetic landscape and also to enhance maturation concomitantly, by bringing artificial alignment reminiscent of natural tissue. Such a strategy could therefore be used in future biomaterials-based approaches in tissue engineering as a means to promote cells differentiation in culture before integration or as part of an integrable system providing mimetic cues that favour maturation [63].
