# Supplementary material for: Enhanced efficiency of genetic programming toward cardiomyocyte creation through topographical cues
Source: Biomaterials. 2015 Nov;70:94–104. doi: 10.1016/j.biomaterials.2015.07.063 (PMC4823279; doi:10.1016/j.biomaterials.2015.07.063)
Supplement: Supplementary file 1 [file mmc1.pdf]

Supporting Information

SI Figure Legends

Figure S1

Clone SP3 on the microgrooved substrate. (A) Actin cytoskeleton (Phalloidin, green), and nuclear (DAPI, blue) staining of SP3 cells cultured for 3 days on flat and patterned substrates (scale bar: 200µm). (B) Scatter plot showing the nuclear elongation (aspect ratio of nuclei elliptical fitting) as a function of each cell's alignment along the grooves (angle between long axis and grooves). Cells on patterned substrates align within 10° parallel to the grooves and are significantly more elongated: 0.52±0.14 on the microgrooved surface versus 0.72±0.13 ($p<10^{-5}$). (C) EdU incorporation analysis showing decreased clone SP3 proliferation on grooves (* $p<0.05$ for Student's t-test, error bars indicate standard deviation, n=3).

Figure S2

Nuclear volume of clone SP16 as evaluated through confocal imaging (20 slices per nucleus) is significantly reduced on the grooved substrates. (*** $p<0.001$; Student's t-test, n=3, error bars indicate standard deviation).

Figure S3

Correlation analysis between nuclear elongation and histone 3 acetylation levels. (A) Scatter plots showing nuclear elongation (y-axis) along histone 3 acetylation (x-axis) and a linear fit (mean-square regression). (B) Average slopes and correlation coefficients between flat and patterned substrates (** $p<0.01$; Student's t-test, n=3, error bars indicate standard deviation). (C) Density plot representing the distribution of histone 3 acetylation levels showing a clear shift toward the right on patterned surfaces.

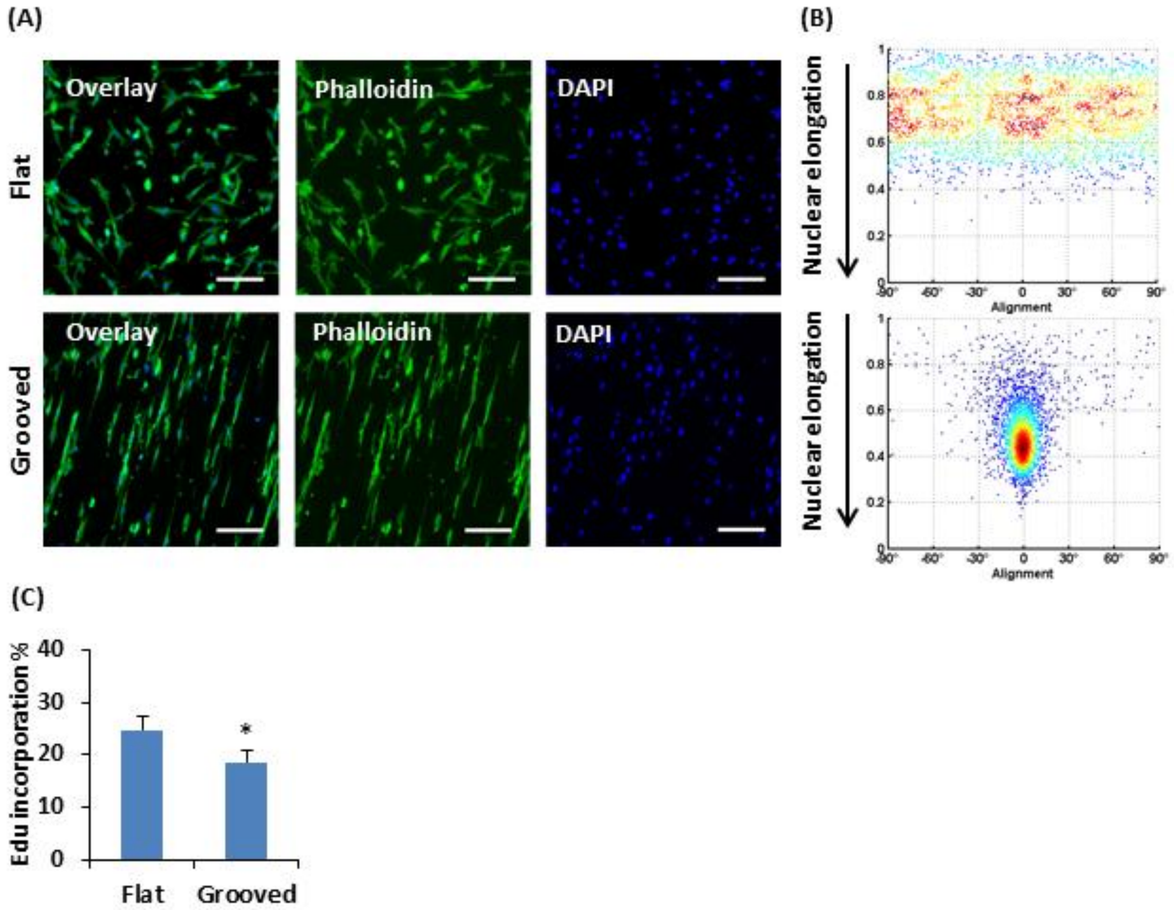

(A)
(B)
Overlay
Phalloidin
DAPI
Flat
Grooved
Nuclear elongation
Alignment
(C)
Edu incorporation %
40
30
20
10
0
*
Flat
Grooved

Figure S1

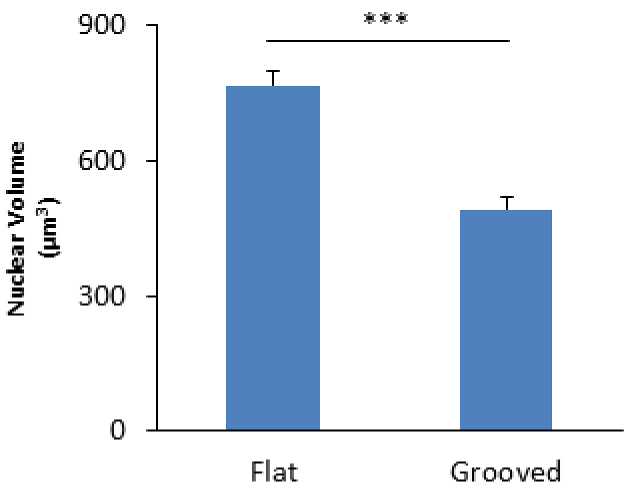

Figure S2

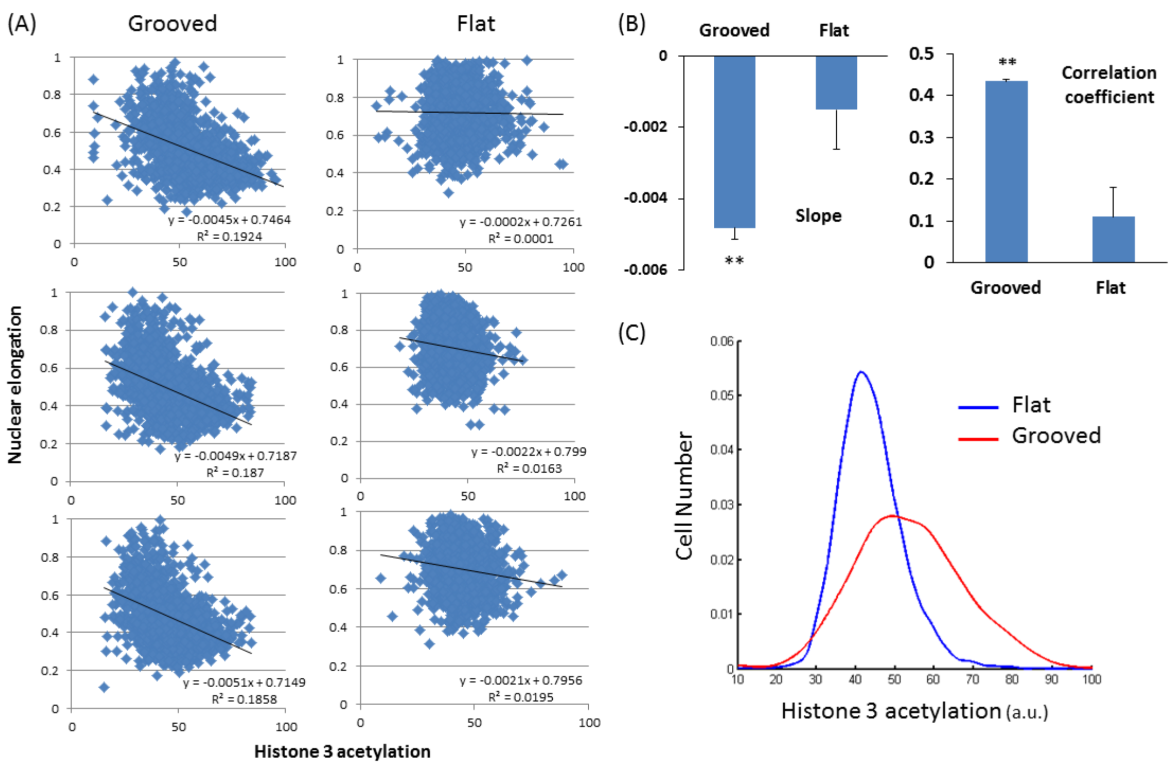
(A)
Grooved
Flat
Nuclear elongation
y = -0.0045x + 0.7464
R² = 0.1924
y = -0.0002x + 0.7261
R² = 0.0001
y = -0.0049x + 0.7187
R² = 0.187
y = -0.0022x + 0.799
R² = 0.0163
y = -0.0051x + 0.7149
R² = 0.1858
y = -0.0021x + 0.7956
R² = 0.0195
Histone 3 acetylation
(B)
Grooved
Flat
Slope
**
Correlation coefficient
**
Grooved
Flat
(C)
Cell Number
Flat
Grooved
Histone 3 acetylation (a.u.)

Figure S3
